# Routine psychosocial distress screening in radiotherapy: implementation and evaluation of a computerised procedure

**DOI:** 10.1038/sj.bjc.6605930

**Published:** 2010-10-26

**Authors:** A Dinkel, P Berg, C Pirker, H Geinitz, S Sehlen, M Emrich, B Marten-Mittag, G Henrich, K Book, P Herschbach

**Affiliations:** 1 Department of Psychotherapy and Psychosomatic Medicine, Division of Psychosocial Oncology, Klinikum rechts der Isar, Technische Universität München, Langerstr. 3, Munich 81675, Germany; 2 IFT-Gesundheitsförderung, Institut für Therapieforschung, München, Munich 80804, Germany; 3 Department of Radiotherapy and Radiooncology, Klinikum rechts der Isar, Technische Universität München, Munich 81675, Germany; 4 Department of Radiotherapy and Radiooncology, Klinikum Großhadern, Ludwig-Maximilians-Universität München, Munich 81377, Germany

**Keywords:** psychosocial distress, distress screening, computerised assessment, psycho-oncology, radiotherapy

## Abstract

**Background:**

To implement distress screening in routine radiotherapy practice and to compare computerised and paper-and-pencil screening in terms of acceptability and utility.

**Methods:**

We used the Stress Index RadioOncology (SIRO) for screening. In phase 1, 177 patients answered both a computerised and a paper version, and in phase 2, 273 patients filled out either the computerised or the paper assessment. Physicians received immediate feedback of the psycho-oncological results. Patients, nurses/radiographers (*n*=27) and physicians (*n*=15) evaluated the screening procedure.

**Results:**

The agreement between the computerised and the paper assessment was high (intra-class correlation=0.92). Patients’ satisfaction did not differ between the two administration modes. Nurses/radiographers rated the computerised assessment less time consuming (3.7 *vs* 18.5%), although the objective data did not reveal a difference in time demand. Physicians valued the psycho-oncological results as interesting and informative (46.7%). Patients and staff agreed that the distress screening did not lead to an increase in the discussion of psychosocial issues in clinician–patient encounters.

**Conclusion:**

The implementation of a distress screening was feasible and highly accepted, regardless of the administration mode. Communication trainings should be offered in order to increase the discussion of psychosocial topics in clinician–patient encounters.

Already at the outset of radiation therapy, many cancer patients experience a reduction in quality of life (Janda *et al*, 2004), reduced physical capacity and pain (Sehlen *et al*, 2001), or emotional distress (Faller *et al*, 2003). Reasonable as well as exaggerated worries about the effects of radiotherapy are not uncommon (Halkett *et al*, 2008), and the psychosocial stress level often remains high, or even increases, during and after radiotherapy (Sehlen *et al*, 2002, 2003b; Chen *et al*, 2009). Studies showed that about 50% of radiotherapy patients suffer from some mental disorder (Leopold *et al*, 1998; Fritzsche *et al*, 2004), underlining the need for psychosocial treatment.

One problem in detecting treatment need is the multitude of possible criteria (Herschbach, 2006). Investigations revealed that between 12 and 43% of radiotherapy patients showed a need for psychosocial treatment, depending on the criteria used (Söllner *et al*, 2001; Faller *et al*, 2003; Fritzsche *et al*, 2004; Hahn *et al*, 2004). In addition, there are further problems in screening and detecting treatment need, which are not limited to radiotherapy. First, the concordance between patient's self-defined treatment need and need defined by oncologists or by symptom checklists is low (Söllner *et al*, 2001; Fritzsche *et al*, 2004; Garssen and de Kok, 2008). Second, oncology professionals are reluctant to apply distress screening instruments, mainly due to lack of time and lack of training (Mitchell *et al*, 2008). Unfortunately, the detection of distress often does not lead to adequate psychosocial treatment (Garssen and de Kok, 2008; Mitchell *et al*, 2008). Finally, there is a lack of studies showing the feasibility and cost-effectiveness of implementing distress screening in routine practice (Kruijver *et al*, 2006; Garssen and de Kok, 2008; Mitchell *et al*, 2008). Despite these difficulties, several agencies promote the routine use of distress screening devices as the primary tool to detect psychosocial treatment need. For example, the American National Comprehensive Cancer Network (NCCN) guidelines recommend that all patients be screened for distress at their initial visit and at intervals thereafter (see Carlson and Bultz, 2003).

In the recent years, several studies investigated the use of computerised screening in oncology, which may be preferable over paper assessment because of convenience and feasibility. Computerised assessment may also circumvent the problem of oncologists’ lack of training in the psychosocial assessment of patients. Studies showed good agreement between computer and paper assessment (Velikova *et al*, 1999), as well as high acceptance and usability, even among older patients and those untrained in using a computer (Allenby *et al*, 2002; Wright *et al*, 2003; Mullen *et al*, 2004; Carter *et al*, 2008). Some studies suggest that computerised assessment is more convenient and time saving than paper assessment (Lane *et al*, 2006; Dale and Hagen, 2007). However, there is a lack of studies on computerised assessment in routine radiotherapy practice. Although some investigations included subsamples of patients undergoing radiotherapy (Allenby *et al*, 2002; Fann *et al*, 2009; Verdonck-de Leeuw *et al*, 2009), to our knowledge there is only one small study that focused exclusively on the radiotherapy setting (Berry *et al*, 2004; Mullen *et al*, 2004).

The aim of this work was to implement a computerised distress screening procedure in routine radiotherapy practice and to compare computerised and conventional paper-and-pencil assessment in terms of acceptability and utility. In contrast to the majority of research, we applied a psychosocial distress screening instrument specifically developed for use in radiotherapy – the Stress Index RadioOncology (SIRO) (Sehlen *et al*, 2003a).

## Materials and methods

### General overview

This was a two-phase study using two independent samples. In the first phase, we investigated the validity of the computerised screening and defined a clinical cutoff score of the SIRO. In the second phase, the screening procedure was implemented in routine radiotherapy care. Patients and staff reported on their satisfaction with the routine screening and the assessment mode. The study was conducted in two university clinics in Munich, Germany. The protocol received approval from the local ethics committee. Table 1 briefly summarises the different steps of this investigation and provides an overview of the assessments used.

### Phase 1 – Validating the computerised assessment

#### Patients

Cancer patients who would be treated with radiotherapy for at least 2 weeks were sampled consecutively for 3 months in two clinics. The inclusion criteria used were as follows: age ⩾18 years old and Karnofsky performance status ⩾50. The exclusion criteria used were insufficient command of the German language, severe cognitive impairment (clinical assessment by the physician), whole body radiation therapy, brachytherapy as the sole treatment modality, stereotactical irradiation, hypofractionated radiotherapy with a treatment duration of less than 2 weeks and participation in a further clinical study. Treatment intent (curative or palliative) did not affect study participation. During 3 months, *N*=180 patients who complied with the criteria were approached, and all agreed to participate. Of those, *n*=177 (98.3%) provided data on the SIRO in both administration modes and therefore represent the final sample. The patients were 58.0 years old on average (s.d.=13.4, range 25–87). The leading diagnoses were breast and prostate cancer (see Table 2).

#### Procedure

Patients were approached after the second radiotherapy fraction within the first treatment week to provide informed consent and to fill out the SIRO. The computerised version was used first and the paper version second in one clinic, and *vice versa* in the other clinic. The time frame for the completion of the SIRO in the two different modalities ranged from 3 to 7 days. During the first of the two assessments, the patients additionally filled out measures of psychological distress. A subsample of patients was assessed with an interviewer-administered distress screening (see Herschbach *et al*, 2008; Siedentopf *et al*, 2010), which will not be reported here.

#### Assessment

The SIRO (Sehlen *et al*, 2003a) was used as a specific psychosocial distress screening. The SIRO measures the current level of perceived stress with 24 items that are rated on a five-point scale from 1 (‘nearly no burden’) to 5 (‘very high burden’), with the additional response option ‘does not apply’. The SIRO comprises four subscales: psychophysical distress, relationship difficulties, radiotherapy-induced distress and information deficits. Reliability of the complete scale is *α*=0.90, and convergent and discriminative validity were established (Sehlen *et al*, 2003a). For the computerised assessment, the items were presented on a tablet-PC. We used Windows XP Tablet PC Edition, and AnyQuest for Windows as software (see www.ql-recorder.com).

Anxiety and depression were assessed with the Hospital Anxiety and Depression Scale (HADS; Hermann-Lingen *et al*, 1995). The two dimensions are measured with seven items per scale. Reliability and validity of the scales were proven in several studies (Hermann-Lingen *et al*, 1995; Olssøn *et al*, 2005).

### Phase 2 – Implementing and evaluating computerised screening

#### Patients

Cancer patients were sampled in the same two clinics as in phase 1. Inclusion and exclusion criteria were the same as in the first phase. Over the course of 6 months, 358 patients who complied with the criteria were asked for study participation; 41 patients declined participation, and 44 patients were excluded as they did not provide an evaluative judgement. Thus, *n*=273 (76.3%) patients represent the final sample. They were 60.4 years old on average (s.d.=11.2; range 19–84); *n*=142 had answered the computerised version, and *n*=131 had filled out the paper assessment. Both groups were comparable with regard to sociodemographic and clinical variables (see Table 2).

#### Staff

Nurses/radiographers (*n*=27) and physicians (*n*=15) provided their evaluations anonymously. Thus, no sociodemographic data are available for the two professional groups.

#### Procedure

*Implementation in routine care*: Patients were approached soonest at their second treatment date. Having provided informed consent, the patients were asked to fill out the SIRO. In one clinic, the computerised version was used for the first 3 months and the paper-and-pencil version for the second 3 months of the study, and *vice versa* in the second clinic. For the paper version, the nurse or the radiographer entered the data in a PC after the patient had answered the SIRO. For the computerised assessment, the data were transferred using bluetooth technology. Next, the results sheet was printed out and deposited in the post box of the treating radiation oncologist. The physician was expected to read the results sheet, which she/he proofed by signature. If the SIRO total score indicated psychosocial distress (easy to realise through a highlighted bar), the treating physician was to call the division for psychosocial oncology for a consultation–liaison (C–L) intervention. The research assistants tracked which of the patients who scored above the cutoff actually received a psycho-oncological intervention.

*Evaluation of the computerised and the conventional assessment*: The patients evaluated the computerised or the conventional paper assessment of the SIRO at least 1 week after their assessment. Those patients for whom psychosocial care was initiated evaluated the SIRO procedure before their first appointment with the C–L psycho-oncologist in order to avoid confounding with the experience of professional support provision. Nurses, radiographers and physicians evaluated the implementation of the routine screening in the final phase of this study, shortly before the collection of the data was terminated.

For both SIRO administration modes, members of the project team took the time nurses/radiographers needed to instruct the patient and the time entering the data and printing out the results sheet. Each nurse/radiographer was observed once handling the computerised assessment and once providing the paper assessment.

#### Assessment

Besides the SIRO, the following measures were applied:

Evaluation of the computerised and the paper assessments was carried out with several evaluative items newly designed for this study:

Patients responded ‘yes’ or ‘no’ to 17 items. Nurses/radiographers and physicians answered 12 items on a five-point scale from ‘completely untrue’ to ‘completely true’. Here, we report only on those items referring to the implementation of the screening procedure, the usability of the two assessment modalities and the satisfaction with the assessment.

Patient satisfaction was assessed with two questionnaires:

Questions on Patient Satisfaction (Fragen zur Patientenzufriedenheit, FPZ; Henrich *et al*, 2001): This questionnaire comprises 10 items relating to different aspects of patient care. The items are rated on a five-point scale ranging from ‘unsatisfied’ (1) to ‘very satisfied’ (5). Each item is also rated with regard to subjective importance, ranging from ‘unimportant’ (1) to ‘extremely important’ (5). A total score is computed by weighing the satisfaction ratings with the respective importance ratings. As some items were inapplicable for outpatients, we used only the four items applicable for in- and outpatients to compute a summary score. These four items mainly focus on health-care staff–patient interaction. The internal consistency of this shortened scale was *α*=80. In addition, the FPZ contains a checklist of 42 possible suggestions for improvement of patient care (e.g. quality of breakfast, waiting times), which are not in the focus of the current report. Investigations showed the validity and utility of FPZ (Henrich *et al*, 2001; Gündel *et al*, 2007).

Satisfaction questionnaire (Patientenfragebogen zur Erfassung der Zufriedenheit, ZUF-8; Schmidt *et al*, 1989): This measure focuses on the satisfaction with the hospital and the care received. It contains eight items that are rated on a four-point scale, with different response options. The items are summed to form a total score. The reliability of this measure in the current sample was *α*=85. Validity has been established (Kriz *et al*, 2008).

#### Statistical analysis

Correlations were computed to measure the convergence between the computerised and the paper assessment of the SIRO. A receiver-operating characteristic (ROC) analysis was carried out in order to establish a cutoff score. We applied descriptive statistics, *χ*^2^ tests and paired sample as well as independent sample *t*-tests using SPSS 15 (SPSS, Chicago, IL, USA).

## Results

### Phase 1

The SIRO was administered without difficulty in both administration modes. The mean of the SIRO total score-paper version (*M*=1.56; s.d.=1.03) did not differ from the SIRO total score-computer version (*M*=1.54; s.d.=0.94); *t* (176)=−0.486, *P*>0.05. The correlation coefficients between the computerised SIRO and its paper assessment counterpart are given in Table 3. The results showed a high convergence between the two versions, with an intra-class correlation (ICC) for the complete scale of *r*=0.92.

The data of the computerised SIRO version were used for the development of the clinical cutoff. Clinical anxiety or depression, assessed with the HADS, represented the criteria against which the SIRO cutoff was established. The mean of the HADS anxiety subscale was *M*=6.23 (s.d.=4.40); the mean of the depression subscale was *M*=5.93 (s.d.=4.80), *n*=175. We used a cutoff ⩾11 for both the HADS anxiety and HADS depression subscale. This cutoff was applied in several studies as indicator of clinically relevant distress (Sellick and Edwardson, 2007; Hinz *et al*, 2010). The ROC analysis suggested a SIRO cutoff of ⩾1.90 (sensitivity 74%, specificity 77%, AUC 0.820). The prevalence of psychosocial distress, applying this cutoff, was 34.5% (*n*=61).

### Phase 2

The mean of the SIRO, across the two administration modalities, was 1.31 (s.d.=0.87). Of the 273 participating patients, 23.8% (*n*=65) scored above the SIRO cutoff and, thus, indicated psychosocial distress. All of these patients should have been referred for the C–L intervention; 73.8% (*n*=48) actually had an appointment with the psycho-oncologist. The rate of the treating physicians who had signed the SIRO results sheet did not differ between the two SIRO administration arms (84.5 *vs* 84.7%).

The patients’ evaluations of the two administration procedures are shown in Table 4. The results reveal high acceptance and good comprehensibility of the SIRO in both administration modes. In total, 7.0 and 5.0%, respectively, of the patients reported that they had talked with the physician about the screening results, and 10.0 and 8.0%, respectively, had talked with the nurse.

Table 5 presents the results of the staffs’ evaluation of the implementation of the distress screening. Of the nurses/radiographers, 18.0% evaluated the paper version as time consuming, as opposed to 3.0% for the computer version. In all, 7.0% acknowledged an increased sensitivity for patients’ psychosocial stress due to the assessment procedures. The physicians found the results sheet comprehensible and interesting, and they showed high acceptance of the computerised assessment. Again, the assessment of the patients’ psychosocial distress did not lead to a marked increase of psychosocial communication topics in the physician–patient communication. Of the physicians, 8.0% stated that they had talked with the patient about the results of the distress assessment in routine interactions.

With regard to the general patient satisfaction, the results obtained with the modified FPZ were significant. The patients who were administered the paper assessment were more satisfied (*n*=120) than the patients who filled out the computerised version (*n*=112); *t* (230)=3.58, *P*<0.001. However, the two groups (*n*=125, *n*=124) did not differ in the ZUF-8 satisfaction rating; *t* (247)=−0.45, *P*>0.05 (see Table 6).

The time demand for the staff (*n*=27) differed for the two steps. Instructing the patient took longer with the computerised administration than with the paper assessment; *t* (26)=3.91, *P*<0.01. In contrast, data handling was faster with the computerised than with the paper assessment; *t* (26)=−8.35, *P*<0.001. However, there was no difference in staff's time demand in total time; *t* (26)=−0.396, *P*>0.05 (see Table 6).

## Discussion

The routine collection of data on patient's psychosocial distress is feasible in routine radiotherapy practice. Our study did not provide clear evidence for the superiority of either one assessment mode. The patients’ evaluations did not differ between the computerised and the conventional paper assessment, both were highly accepted. However, the nurses/radiographers tended to rate the computerised version less time consuming. However, even for the paper assessment, only a minority stated that the assessment procedure was too long.

Despite these positive evaluations, the nurses/radiographers did not experience a positive impact of the distress screening on the discussion of patients’ psychosocial issues in the team. Physicians stated heightened personal interest in patients’ psychosocial issues due to the distress screening. However, they also reported that the distress screening itself and the screening results generally did not enter the realm of physician–patient communication – an observation that was shared by the patients.

This result of our study adds to the slowly accumulating evidence about the clinical consequences of the assessment of distress and patient reported outcomes (PRO). Some recent reviews in oncology (Kruijver *et al*, 2006; Luckett *et al*, 2009) and other fields of medicine (Greenhalgh *et al*, 2005; Valderas *et al*, 2008) conclude that feedback of PROs has little impact on patient management and patient outcomes. There seems to be some positive effect of PRO feedback on physician–patient interaction, however. In one of those studies in routine oncology practice (Velikova *et al*, 2004), which showed a positive impact on communication, physicians were trained in interpreting quality of life data. Furthermore, they were encouraged to make use of the information during all encounters. In our study, the physicians were instructed to call the psycho-oncologist in case of treatment need, as assessed by the distress screening procedure. Therefore, the physicians might have felt less committed to the idea of discussing the results of the distress screening, regardless of its administration mode. Thus, our results tentatively suggest that with increasing specialisation of care, there is the risk of increasing division of labour and diffusion of responsibility.

It seems to be desirable that oncologists communicate with the patient about the results of a distress screening, even if the patient is referred to C–L psycho-oncological care. However, in this case, there is the need for an explicit agreement and plan of action between the parties involved. It might even be desirable that oncologists use the feedback in case of absent increased distress in order to reinforce the patient's successful coping. Some data show that the use of PRO measures in managing patients does not increase consultation length (Luckett *et al*, 2009), but this can only be satisfactorily achieved if the physician can rely on good communication skills.

With regard to the validity and technical issues of the computerised and the conventional paper assessment of the SIRO, our results are in line with most of the available research. There was high congruence between the two assessment modes, and it can be concluded that mode of administration does not make a difference with regard to the reliability and validity of the SIRO (see Gwaltney *et al*, 2008; Velikova *et al*, 1999). The total duration of assessment turned out to be quite identical for both modalities. Finally, the two screening modalities did not differentially impact on the patients’ general satisfaction with the hospital and the care received, but there was a difference on the more specific measure of patient satisfaction with care (modified FPZ). To speculate, the patients who filled out the paper assessment might have expressed higher satisfaction with care as they experienced more interaction with staff and a less ‘technical’ atmosphere when filling out the SIRO.

Limitations of our study pertain to the research design, as only a randomised controlled trial would provide strong evidence concerning differential utility of the two assessment modalities. Furthermore, we did not ask the patients about their familiarity with computers. However, the patients indicated no problems in using the touch screen.

To conclude, the implementation of a routine distress screening with immediate feedback to the physician was feasible and highly accepted and resulted in high referral rates to a C–L psycho-oncological intervention. Computerised assessment was valued by the professionals, but it was not preferred above the conventional paper assessment by the patients. As the distress screening and the feedback of the results did not lead to an increase in the communication about psychosocial issues in the clinician–patient encounter, it seems necessary to offer communication trainings for clinicians (see Rodin *et al*, 2009), or to implement clinical supervision in order to strengthen reflective clinical practice.

## Figures and Tables

**Table 1:** General overview of the study

**Sample**	**Topic**	**Assessments**
*Phase 1*		
177 patients	Concordance computerised and paper-and-pencil assessment	SIRO computerised and paper-and-pencil (each patient both assessment modes)
	Defining clinical cutoff score for screening instrument	HADS
		
*Phase 2*		
142 patients	Distress screening	SIRO computerised
131 patients	Distress screening	SIRO paper-and-pencil assessment
		
273 patients (whole sample)	Evaluation screening procedure	Newly designed evaluative items
	Patient satisfaction	FZP and ZUF-8
		Newly designed evaluative items
		
27 Nurses/radiographers	Evaluation screening procedure	Newly designed evaluative items
		
15 Physicians	Evaluation screening procedure Time demand for assessment	Newly designed evaluative items

Abbreviations: SIRO=Stress Index RadioOncology; HADS=Hospital Anxiety and Depression Scale; FZP=Questions on Patient Satisfaction; ZUF-8=Satisfaction Questionnaire.

**Table 2:** Sociodemographic characteristics of the study samples

	**Phase 1 sample**		**Phase 2 sample**					
			**Whole sample**		**Paper-and-pencil assessment**		**Computerised assessment**	
	**(*n*=177)**		**(*n*=273)**		**(*n*=131)**		**(*n*=142)**	
	***M***	**s.d.**	***M***	**s.d.**	***M***	**s.d.**	***M***	**s.d.**
Age (years)	58.0	13.4	60.4	11.2	60.2	11.2	60.6	11.3
								
	***n***	**%**	***n***	**%**	***n***	**%**	***n***	**%**
*Sex*								
Female	87	49.2	151	57.4	80	61.1	71	53.8
*Living situation*								
Alone	50	28.2	74	28.2	44	33.6	30	22.9
With partner	127	71.8	188	71.8	87	66.4	101	77.1
*Cancer site*								
Breast	53	30.1	91	35.0	44	34.1	47	35.9
Prostate	34	19.3	57	21.9	21	16.3	36	27.5
Head/neck	24	13.6	22	9.2	13	10.1	9	6.9
Brain	19	10.8	13	5.0	5	3.9	8	6.1
Gastrointestinal	10	5.7	18	6.9	11	8.5	7	5.3
Other	36	20.5	59	22.7	35	27.1	24	18.3
*Disease state*								
Disease free	20	11.4	31	12.1	13	10.3	18	13.7
First occurrence	113	64.2	159	61.9	78	61.9	81	61.8
Recurrence	17	9.7	29	11.3	12	9.5	17	13.0
Metastases	9	5.1	21	8.2	16	12.7	5	3.8
Second neoplasm	17	9.7	17	6.6	7	5.6	10	7.6

*Note*: Sample size may differ owing to missing data.

**Table 3:** Correlations between the computerised and the paper-and-pencil version of the SIRO (*n*=177); Pearson (*r*) and ICC

**Scale**	***r***	**ICC**
Psychophysical distress	0.86	0.92
Relationship difficulties	0.73	0.84
Radiotherapy-induced distress	0.82	0.90
Information deficits	0.78	0.87
Total score	0.86	0.92

Abbreviations: SIRO=Stress Index RadioOncology; ICC=intra-class correlations.

**Table 4:** Patients’ (*n*=113–131) responses to the items evaluating the assessment procedures; percentage (%) of patients agreeing (‘yes’) with the statement

**Evaluative items**	**Paper and pencil**	**Computerised**	***P*-value**
I understood easily how to fill out the questionnaire	96.9	99.2	NS
The font size was easily readable	99.2	94.7	NS
The questionnaire was too long	20.5	12.1	NS
I felt uncomfortable providing personal information	9.5	14.1	NS
I felt some time pressure when filling out the measure	6.3	7.1	NS
The writing on the screen was well perceptible		99.2	—
The touch-screen computer was too heavy, too unwieldy		0	—
I was well instructed on how to handle the computer		98.4	—
Did you get the impression that your physician referred to the contents of the questionnaire?	16.5	19.3	NS
Did you get the impression that your physician took more time for your encounters?	41.0	35.6	NS
Did you get the impression that the physician had an increased interest in your emotional state?	35.9	26.3	NS
Did you get the impression that the physician had prescribed further treatments?	27.2	19.5	NS
Did the physician discuss the results with you?	7.3	4.9	NS
Did the nurses refer to the results?	10.4	7.9	NS
Would you prefer to get psycho-oncological treatment due to the results of the questionnaire?	31.1	32.5	NS

Abbreviation: NS=not significant.

**Table 5:** Nurses/radiographers' (*n*=27) and physicians' (*n*=13–15) evaluation of the screening procedures; mean (*M*), standard deviation (s.d.) and percentage (%) agreement (responses ‘4’ and ‘5’, high agreement)

**Evaluative items**	***M***	**s.d.**	**%**
*Nurses/radiographers*			
The time spent with the paper version was too long	2.3	1.3	18.5
The time spent with the computerised version was too long	1.5	0.8	3.7
I felt uncomfortable in the initial interaction orienting the patient	1.3	0.7	3.7
The data entry for the paper version was too time consuming	2.4	1.2	18.5
The screening procedure is an additional burden for the patients	1.9	0.8	0.0
The sensitivity regarding the subjective experiences of the patients has increased in our team due to this project	2.1	1.0	7.4
Actually, conversations with colleagues now more often refer to the emotional state of the patients	2.2	1.1	7.4
			
*Physicians*			
The psycho-oncological findings were readable and clear	4.5	0.7	93.3
The psycho-oncological findings were interesting and informative	3.5	1.2	46.7
I already knew the results of the psychosocial assessment before	2.9	1.0	20.0
In some cases I introduced additional treatments owing to the psycho-oncological results	2.6	1.3	28.6
Somehow I have become more attentive to the subjective emotional state of the patients	2.8	1.5	35.7
I think that the computerised assessment is an unreasonable demand for the patients	2.1	1.0	6.7
I referred to the psycho-oncological results when talking to the patients	2.7	1.1	7.7
The visit takes more time now then before the project had started	1.7	0.9	15.4
I referred to the psycho-oncological results in patient visits	2.1	0.9	15.4

*Note*: The items were rated on a five-point scale from ‘completely untrue’ (1) to ‘completely true’ (5).

**Table 6:** General patient satisfaction and staff time demand for conducting SIRO computerised *vs* paper-and-pencil assessment

	**SIRO computerised assessment**		**SIRO paper assessment**		
	***M***	**s.d.**	***M***	**s.d.**	***P*-value**
Modified FPZ	11.1	4.9	13.3	4.5	<0.001
ZUF-8	28.5	3.2	28.3	3.2	NS
					
*Time demand (min)*					
Instructing	4.21	3.32	1.46	0.51	<0.01
Data handling	1.56	1.08	4.56	1.54	<0.001
Total	6.19	4.07	6.34	2.09	NS

*Abbreviations*: FPZ=Questions on Patient Satisfaction; ZUF-8=Satisfaction Questionnaire; SIRO=Stress Index RadioOncology; NS=not significant.
